# P2X7 receptor antagonism modulates IL-1β and MMP9 in human atherosclerotic vessels

**DOI:** 10.1038/s41598-017-05137-y

**Published:** 2017-07-07

**Authors:** Maria Lombardi, Maria Elena Mantione, Domenico Baccellieri, David Ferrara, Renata Castellano, Roberto Chiesa, Ottavio Alfieri, Chiara Foglieni

**Affiliations:** 1 Cardiovascular Research Area, IRCCS San Raffaele Scientific Institute, Milano, Italy; 2 Cardio-thoracic-vascular Department, IRCCS San Raffaele Scientific Institute, Milano, Italy

## Abstract

In atherosclerosis, matrix metallopeptidases (MMPs) contribute to plaque rupture through weakening of the fibrous cap. Pleiotropic P2X purinoceptor 7 (P2X7), expressed in the carotid plaque (PL), is involved in interleukin 1 beta (IL-1β) release that may influence MMP9 generation, thus their possible modulation through acting on P2X7 was investigated. P2X7-related machinery was characterized and the effects of P2X7 antagonists (A740003, KN62) and MMPs inhibitors (Batimastat, Ro28-2653) were studied in *ex-vivo* tissue cultures of human PL’s vs. non-atherosclerotic internal mammary artery (IMA) by using molecular biology, immune-biochemical and microscopy methodologies. We highlighted atherosclerosis-related differences between PLs and IMAs molecular patterns, and their responsivity to P2X7 antagonism. High IL-1β tissue content was associated with PLs morphology and instability/vulnerability. We demonstrated that A740003, but not KN62, decreased IL-1β and MMP9 independently from NLR family pyrin domain containing 3, but in relationship with patient’s smoking status. Acting downstream P2X7 by MMPs inhibitors, diminished IL-1β mRNA without transcriptional effect at MMP9, possibly because the assumption of statin by patients. These data firstly demonstrated A740003 suitability as a specific tool to decrease inflammatory status in human vessels and might support the design of studies applying P2X7 antagonists for the local targeting and tailored therapy of atherosclerosis.

## Introduction

Atherosclerotic plaque destabilization remains unpredictable and the identification of strategies to stabilize the lesion represents a challenge.

Loss of the extracellular matrix is involved in destabilization, and matrix metallopeptidase 9 (MMP9) may affect the overall plaque stability^1, 2^. MMP9 yields to caspase-1 -independent activation of the pro-inflammatory cytokine interleukin 1 beta (IL-1β)^3^, a gatekeeper of inflammation^4^. In turn IL-1β produced by plaque endothelium, smooth muscle cells, and monocytes/macrophages, increases the expression and the activity of MMP9 via activation of different pathways^5, 6^.

Rapid release of MMP9 has been related to ATP-activated P2X purinoceptor 7 (P2X7), in circulating cells^7^. After the demonstration that nucleotides not only have a physiological energetic function but also play a pathological role^8^, the interest in studying purinergic receptors is therefore growing rapidly. Compelling evidence has been published implicating that ATP-triggered P2X7 pathway in the IL-1β processing/release, is mediated either via microvesicles or lysosomes-involving secretory pathways^9, 10^.

P2X7 is more expressed in carotid arteries bearing plaques than in non-atherosclerotic arteries^11^, thus it could play a role in the IL-1β-dependent regulation of MMP9 and ultimately in the modulation of plaque destabilization. Acting on P2X7 might affect the vascular levels of IL-1β and of MMP9. We used an *ex-vivo* tissue culture model^11^ of human carotid plaque (PL) and internal mammary artery (IMA), which is susceptible to a mild inflammation^12^ but not prone to atherosclerosis) for studying P2X7-related machinery. Modulation of IL-1β and MMP9 gene expression, molecular content, activity and release was investigated in relation to PL instability. Here we demonstrated for the first time in *ex-vivo* human vessel tissue cultures the post-transcriptional efficacy of P2X7-specific antagonist, A740003, in decreasing vascular inflammation and the implications of a patient’s smoking status. These results provide a basic framework for the application of A740003 or other P2X7 specific antagonists as a tool to decrease inflammatory status of arteries, in the perspective of stabilizing atherosclerotic plaques. Further studies will validate their applicability for a therapeutic local -targeting aimed to reduce cardiovascular risk on the top of the standard care^13, 14^.

## Results

### PL and IMA: Differences in P2X7-related machinery

**Figure 1:**
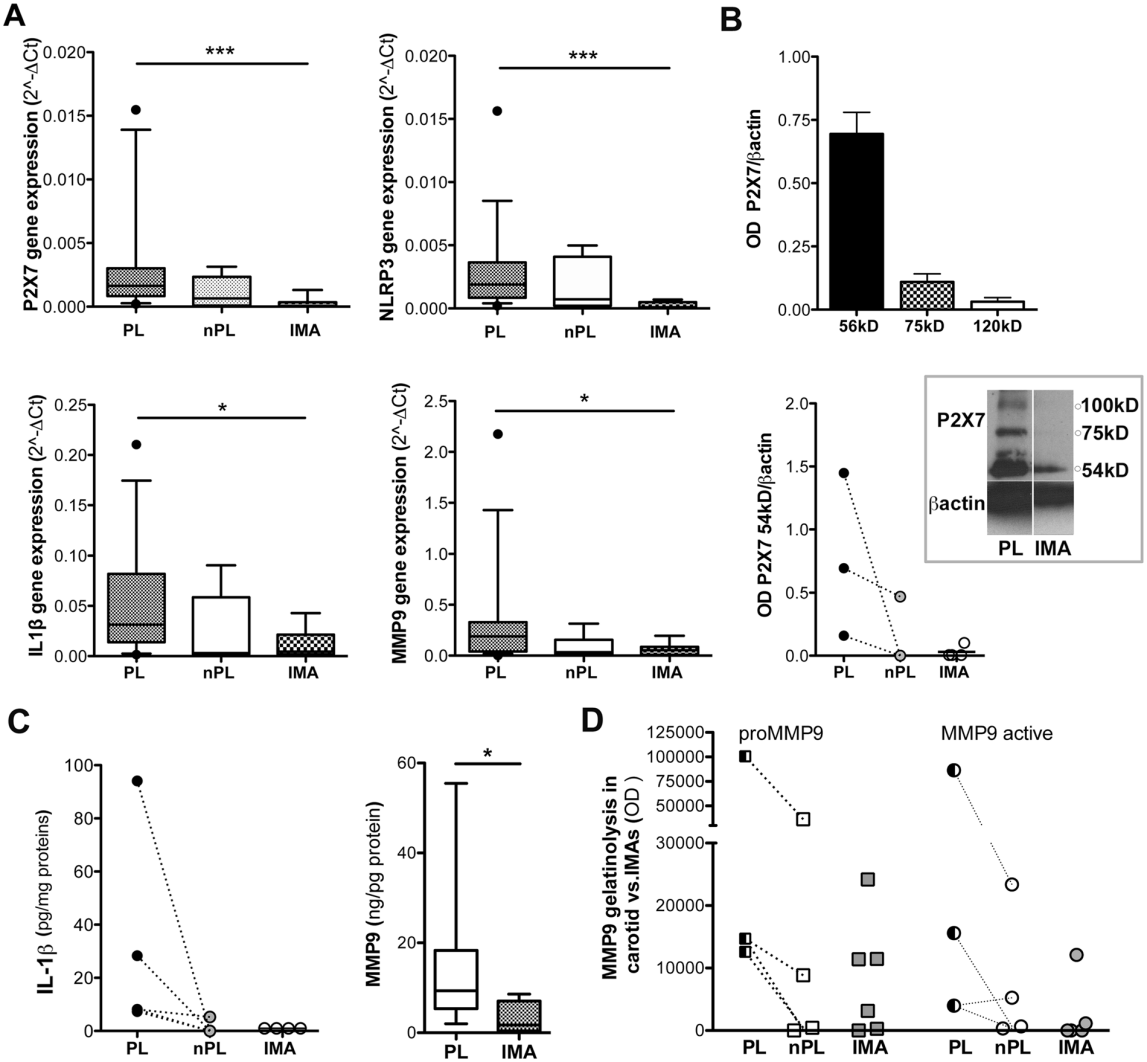
P2X7 - MMP9 machinery in *ex-vivo* culture of carotid and internal mammary arteries. P2X7, NLRP-3, IL-1β, MMP9 mRNA expression by real-time PCR in IMAs, nPL and PL fragments is shown (**A**). Quantification of P2X7 isoforms expression in PL (**B**, top graph) and of 54 kD isoform in PL, nPL and IMA **(B** bottom graph) by OD densitometric analysis is presented; a representative cropped western blot showing P2X7 in PL and in IMA is in the inset. Tissue content of IL-1β (**C** left) and MMP9 (**C** right) in protein extracts from arteries is presented. MMP9 content is measured in IMAs (n = 4) and PLs (n = 22). Optical density (OD) analysis of MMP9 gelatinolytic activities for PL, nPL and IMA is shown (**D**). Points with before –after connecting lines indicate fragments from the same vessel. Values are presented as bars ± SEM, or boxes with 5–95 percentile (• indicates outlier). Kruskal-Wallis and Dunn’s Multiple Comparison tests are applied. Significant differences are shown as *p < 0.05 and ***p < 0.001.

A P2X7-related vascular profile, either caspase-1/NLRP3-dependent or independent could be involved in IL-1β activation and in MMP9 regulation. To depict the possible differences between atherosclerotic and non-atherosclerotic arteries, *ex-vivo* static tissue cultures of PL, of carotid fragments non-bearing atheroma (nPL) and of IMA rings were compared. The expression of P2X7, IL-1β, NLRP3 and MMP9 mRNAs were higher in PLs than in nPLs and IMAs (Fig. 1A), and moderate correlation of expression levels was found in PLs (Table I).

PLs expressed P2X7 isoforms at 54 kD, 75 kD and >100 kD in variable extent, while IMAs and nPLs expressed only P2X7 at 54 kD (Fig. 1B). Scattered P2X7^+^ cells along the lumen and traces of signal in the walls were observed in IMA sections. According to previous findings^11^, P2X7 fluorescence was detected at luminal site, into both neointima and media of PLs sections (Supplementary Fig. IA), and demonstrated a granular pattern in αSMA^+^ and CD68^+^ cells, as well as in the surrounding interstitial space (Supplementary Fig. IB).

IL-1β was detected by ELISA assay in PL tissues, absent or present in negligible quantity in nPLs and IMAs, and in all the culture supernatants (Fig. 1C left, Supplementary ﻿Fig. IC)). Western blot demonstrated the inactive form of IL-1β (pro IL-1β) in PLs extracts only (Supplementary Fig. ID left). NLRP3 and caspase-1 proteins were detected in atherosclerotic and non-atherosclerotic arteries (Supplementary Fig. ID right).

According to MMPs involvement in atherosclerosis, the MMP9 content (Fig. 1C right) and gelatinolytic activities (Fig. 1D) were higher in extracts from PL tissues than in those from non-atherosclerotic arteries. MMP9 released by PLs into culture supernatants was also higher than that of IMAs (Supplementary Fig. IE).

No relationship could be established between vascular samples and clinical data of patients.

These findings, delineating two P2X7 machinery patterns for atherosclerotic and non-atherosclerotic arteries, respectively, supported the use of both nPL and IMA as suitable control for PL.

### PL instability and P2X7-related machinery

**Figure 2:**
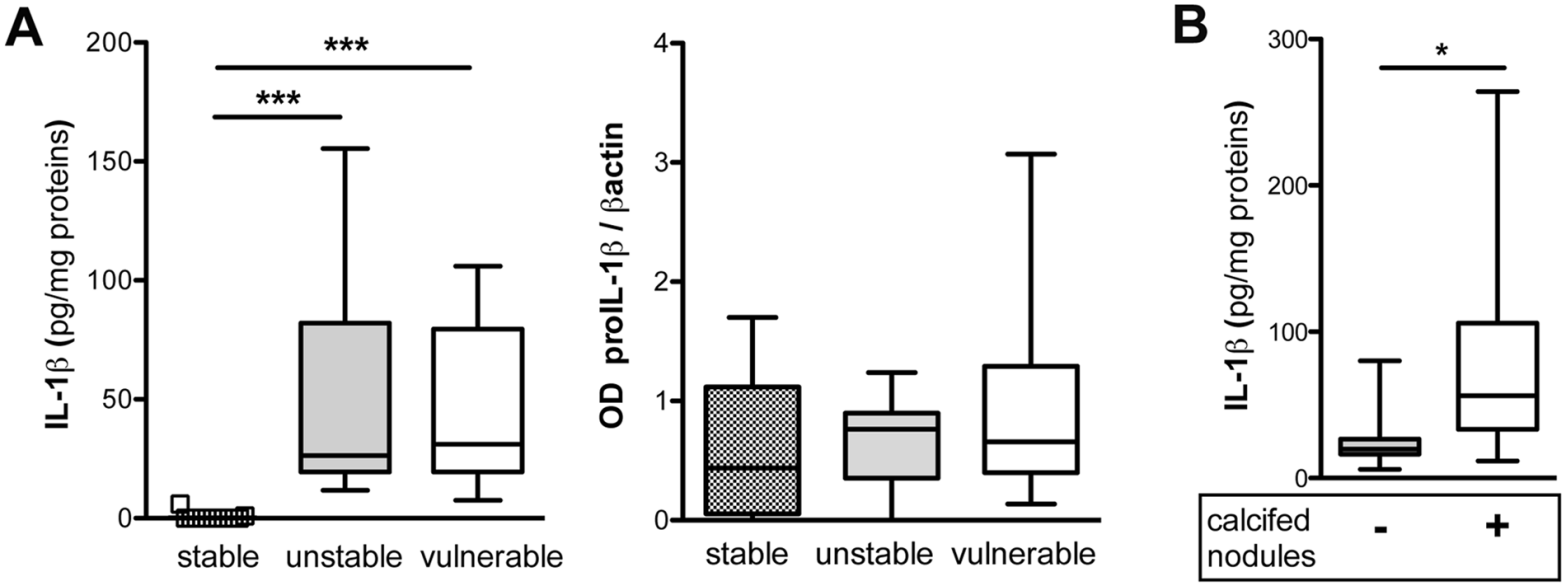
L-1β content and PL instability. Comparison of IL-1β content in protein extracts from unstable (n = 13) vulnerable (n = 15) and stable (n = 15) PLs is shown (**A**, left graph). Quantification of proIL-1β by OD densitometric analysis is displayed (**A**, right graph). Kruskal-Wallis and Dunn’s Multiple Comparison tests are applied. Relationship between high IL-1β amount and the presence of calcified nodules underneath the fibrous cap of PLs is presented (**B**). Unpaired t-test is applied. Values are presented as boxes with 5–95 percentile, significant differences are shown as *p < 0.05 and ***p < 0.001.

The connection between the activated status of P2X7-related machinery and the plaque instability was evaluated in PLs tissue cultures. IL-1β was not or barely detected (0 ≤ IL-1β < 6 pg/mg protein) in extracts from stable PLs, but measured in discrete amounts (10 pg/mg protein < IL-1β < 260 pg/mg protein) in those from unstable and vulnerable PLs (Fig. 2A left). The expression of pro-IL-1β in PLs did not differ in the three categories (Fig. 2A right) suggesting the involvement of active IL-1β in plaque destabilization.

IL-1β content was highest in extracts from PLs with calcified nodules underneath the fibrous cap (p = 0.0161, Fig. 2B). No other relationship to PLs morphology was found, highlighting the association of IL-1β with PLs destabilization.

### P2X7 antagonism effects on IL-1β in *ex-vivo* vessel cultures t1

To evaluate the possible role of P2X7 antagonism on vascular inflammatory status, *ex-vivo* arteries were treated for 24 h (t1) either with A740003, a selective P2X7 antagonist, or with KN62, a potent inhibitor of CamKII and non-competitive P2X7 antagonist.

**Figure 3:**
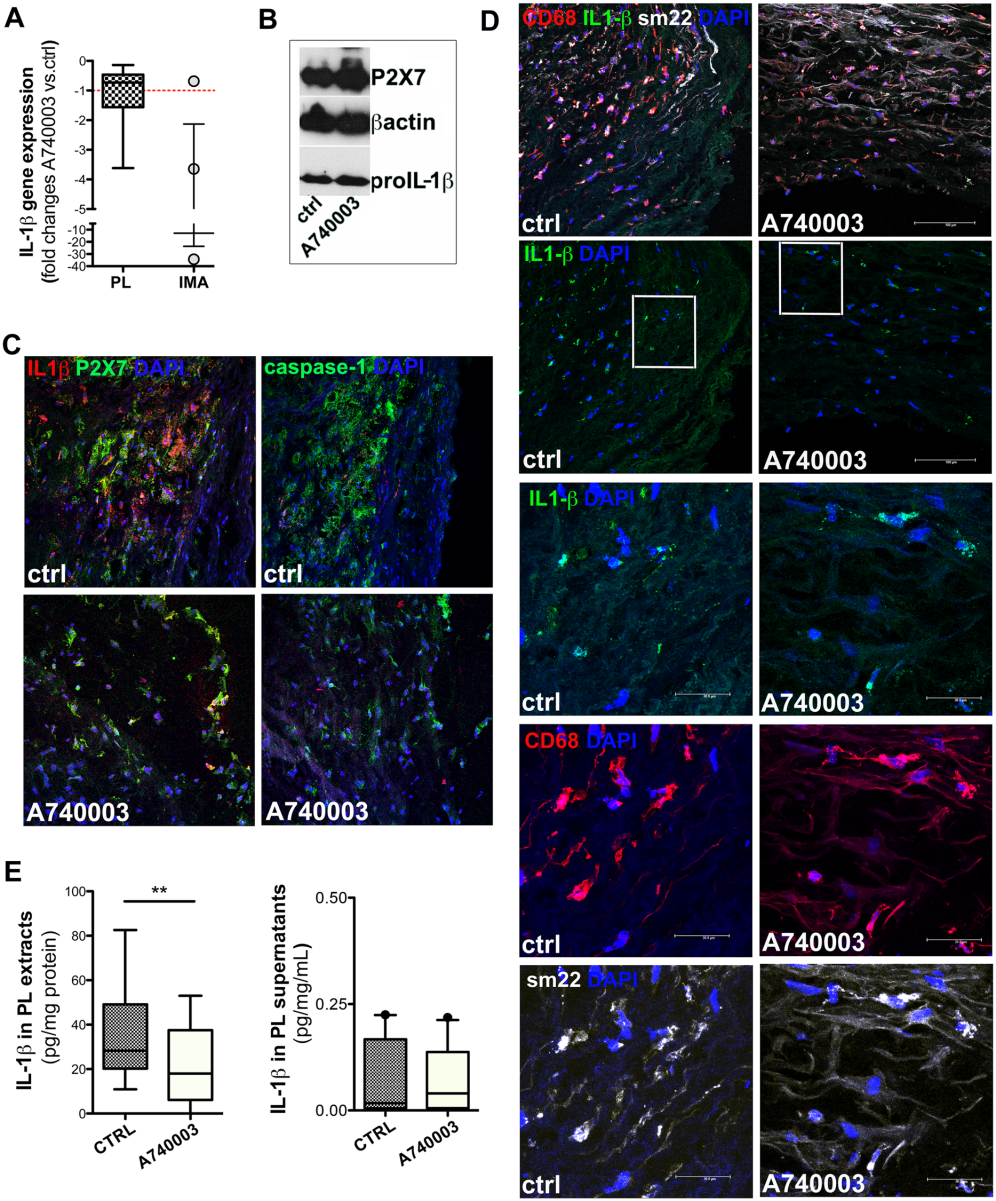
*Ex-vivo* vessels culture at t1: A740003 effects on IL1β. IL-1β gene expression at t1 in PLs (n = 15) and IMA (n = 3) treated with A740003 (**A**) is shown. Red dotted line indicates ctrl value. Representative cropped western blot shows P2X7 and pro-IL1β expression in PLs protein extracts (**B**). Representative confocal microscopy 2D free projection max images from PLs intima are in (**C** and **D**). In (**C**) P2X7 (green), IL-1β (red) and caspase-1 (green) are shown. In **D** upper row CD68 (red), IL-1β (green) and sm22 (white) signals are merged. In (**D**) 2^nd^ row is IL-1β and a white square indicates the regions magnified (3x zoom) in (**D**) 3–5^th^ rows. Nuclei are stained with DAPI (blue). IL-1β content in extracts (n = 25) and supernatant from PL (n = 23) untreated/treated with A740003 (**E**) is displayed. Values are shown as boxes with 5–95 percentile (• indicates outlier). Paired t-test was used. Significant difference is shown as **p < 0.01.

Neither mRNA levels nor expression of pro-IL-1β and P2X7 proteins did significantly change in PLs and IMAs treated with P2X7 antagonists with respect to untreated rings (ctrl) (Fig. 3A, B and Fig. IIA). At difference, qualitative evaluation by confocal microscopy displayed a poorer IL-1β signal in the intima of PLs treated either with A740003 (Fig. 3C) or with KN62 (Supplementary Fig. IIB) than in ctrl. Treatment with A740003 reduced IL-1β mostly into CD68^+low^/sm22^+low^ cells and in the surrounding interstitial space, and in a lesser extent on CD68^+high^/sm22^+high^ cells. This observation was supported by ELISA quantitative analysis showing a significant decrease of IL-1β content in extracts from PLs treated with A740003 vs. ctrl (p = 0.0083, Fig. 3D left), not due to protein release into supernatants (Fig. 3D right). Comparison of IL-1β measurement in a subset of PLs treated in parallel with the two P2X7 antagonists (Supplementary Fig. IIC) showed a more variable response to KN62 than to A740003. The absence of significant efficacy of KN62 in decreasing IL-1β content was retained when the sampling was increased (Supplementary Fig. IID,E).

These data indicated a role for specific P2X7 antagonism in modulating IL-1β production into atherosclerotic arteries.

To exclude that P2X7 antagonists increased extracellular ATP through binding impairment, thus on one side they diminished the levels of an inflammatory cytokine but on the other contributed to create a pro-inflammatory environment, the ATP content was measured. ATP did not accumulated into IMA and in PLs treated with A740003 or KN62 (Supplementary Fig. IIIA), and it was released into supernatant comparably to ctrl (Supplementary Fig. IIIB). Results indicated that no negative side effect on ATP followed the treatments with P2X7 antagonists.

### Effect of treatment with A740003 on IL-1β in *ex-vivo* vessel cultures at t3

**Figure 4:**
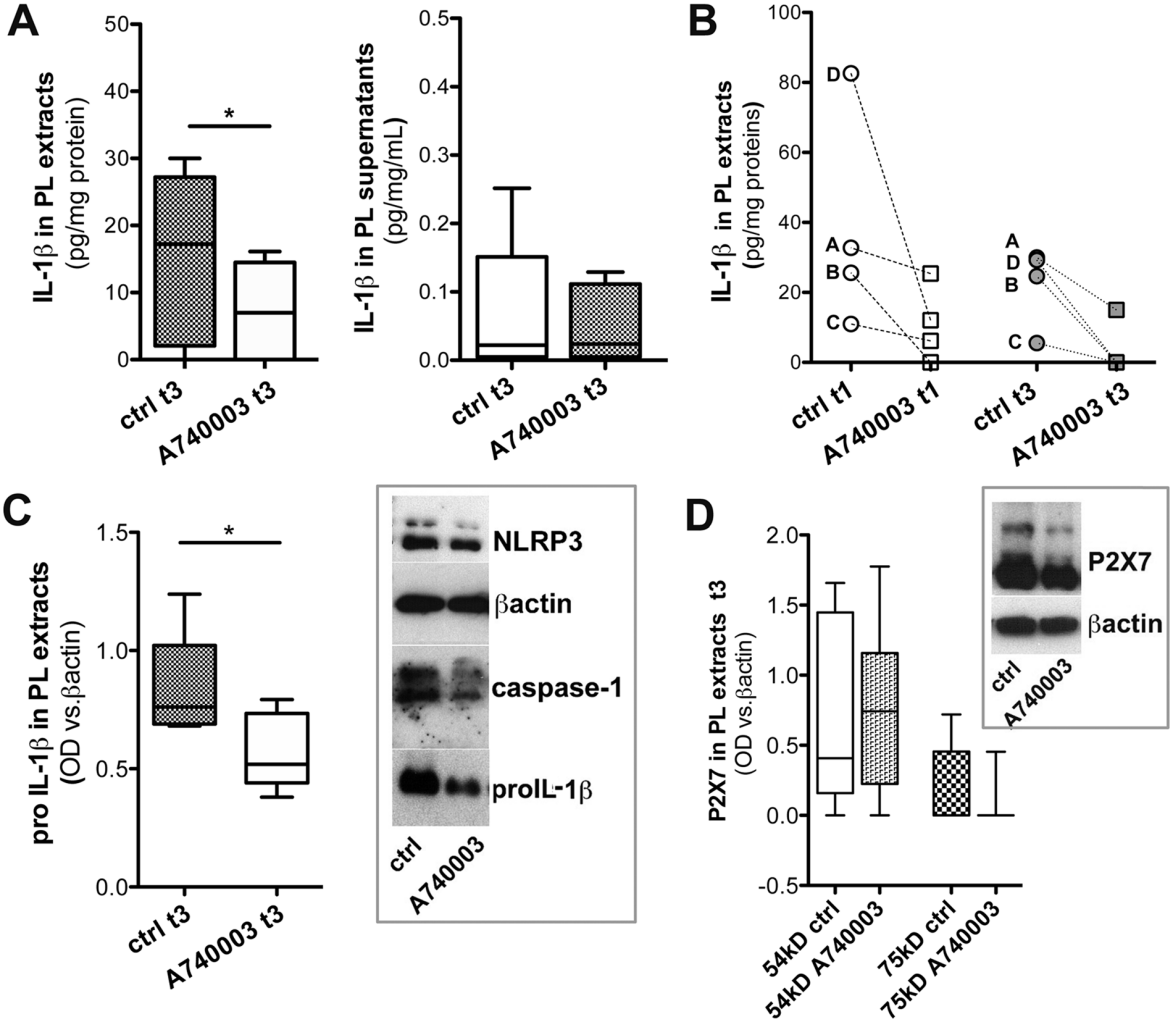
*Ex-vivo* vessels culture at t3: A740003 effects on IL1β. IL-1β content in PL tissues and culture supernatants after treatment with A740003 at t3 are shown (**A**). IL-1β content in a subset of PLs which fragments were treated for 24 h (t1) and 72 h (t3) with A740003 are shown. Points with before–after connecting lines and letters indicate fragments from the same vessel. (**B**). Quantification of proIL-1β by OD densitometric analysis in PLs treated with A740003 at t3 (**C**, left) and a representative cropped western blot of P2X7-machinery (**C**, right) are shown. Densitometry of P2X7 isoforms at 75 kD and 54 kD in PLs treated with A740003 at t3 is in (**D**) with representative cropped western blot in the inset. Values displayed as boxes are presented with 5–95 percentile. Paired t-test is used; significant difference is shown as *p < 0.05.

A740003 potentiality as a modulator of P2X7-related machinery was further investigated in *ex-vivo* vessel cultures treated for 72 h (t3). Similarly to t1, in PLs treated with A740003 no morphology alteration or difference in mRNA levels was observed vs. ctrl (Supplementary Fig. IIIC,D), but a decrease in IL-1β tissue content (p = 0.0249) without increase of the cytokine into supernatants (Fig. 4A) was found. IL-1β determination in the PLs’ subset treated with A740003 at both t1 and t3 highlighted these findings (Fig. 4B).

At t3 pro-IL-1β expression significantly decreased in PLs treated with A740003 (p = 0.0222) (Fig. 4C), while P2X7, NLRP3 and caspase-1 were not appreciably modified (Fig. 4C,D).

These data supported that A740003 post-transcriptionally modulated IL-1β in *ex-vivo* PLs, without involving NLRP3/caspase-1 pathway.

### P2X7 antagonism effects on MMP9 in *ex-vivo* vessel cultures

**Figure 5:**
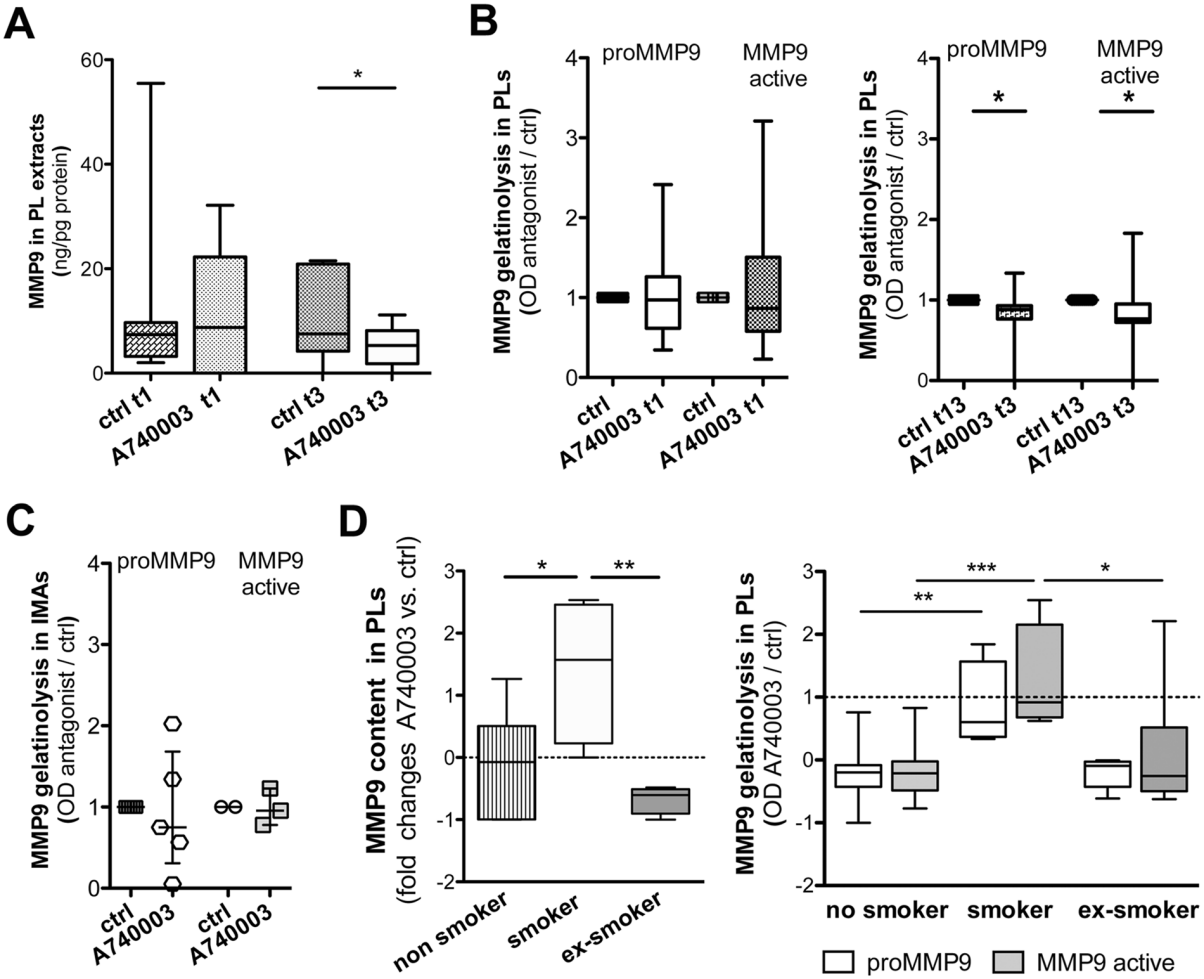
*Ex-vivo* vessels culture: A740003 effects on MMP9 content and activities. MMP9 content in PL extracts after treatment with A740003 is displayed (**A**). Paired t-test is applied. Quantification of MMP9-related gelatinolytic activities in PL extracts after treatment with A740003 at t1 and t3 is plotted (**B**). Wilcoxon signed rank test is applied. MMP9-related gelatinolytic activities in IMA extracts are presented (**C**) as scatter dot plot with median and interquartile range. A740003 on patients grouped for their smoking status: efficacy in diminishing the MMP9 content and activities is shown (**D**). Dotted line displays the control value in (**D**) histograms. One-way ANOVA with Bonferroni post-hoc test is used. Values displayed as boxes are shown with whiskers 5–95 percentile. Significant differences are shown as *p < 0.05, **p < 0.01 and ***p < 0.001.

At t1 the treatment with P2X7 antagonists was ineffective on MMP9 content and gelatinolytic activities in *ex-vivo* vascular tissues (Fig. 5A,B left) and supernatants (Supplementary Fig. IV).

At t3 a significant decrease of MMP9 amount (p = 0.0442) (Fig. 5A) and of pro-MMP9 gelatinolytic activity (p = 0.0322) (Fig. 5B right) was measured in the PLs treated with A740003 vs. ctrl, paralleled by a moderate increase of MMP9 active gelatinolytic activity into supernatants (p = 0.0488) (Supplementary Fig. IVD right). In IMAs treated with A740003 vs. ctrl no changes were observed (Fig. 5C).

These results indicated that A740003 could modulate MMP9 in PLs only after 72 h of treatment, possibly due to the difficulty of acting downstream IL-1β.

### P2X7 antagonist efficacy on MMP9 and smoke

Grouping patients by smoking status evidenced the efficacy of treatment with A740003 in decreasing MMP9 content and gelatinolytic activities in PLs from non-smokers and ex-smokers. In PLs from smokers the opposite phenomenon was found (Fig. 5D). No relationship was observed with IL-1β content or other molecules expression, which suggest that smoking status was negatively associated to the feasibility of MMP9 modulation via P2X7 antagonism.

### MMP9 inhibitors: effect on IL-1β and P2X7-related machinery in *ex-vivo* vessel cultures

**Figure 6:**
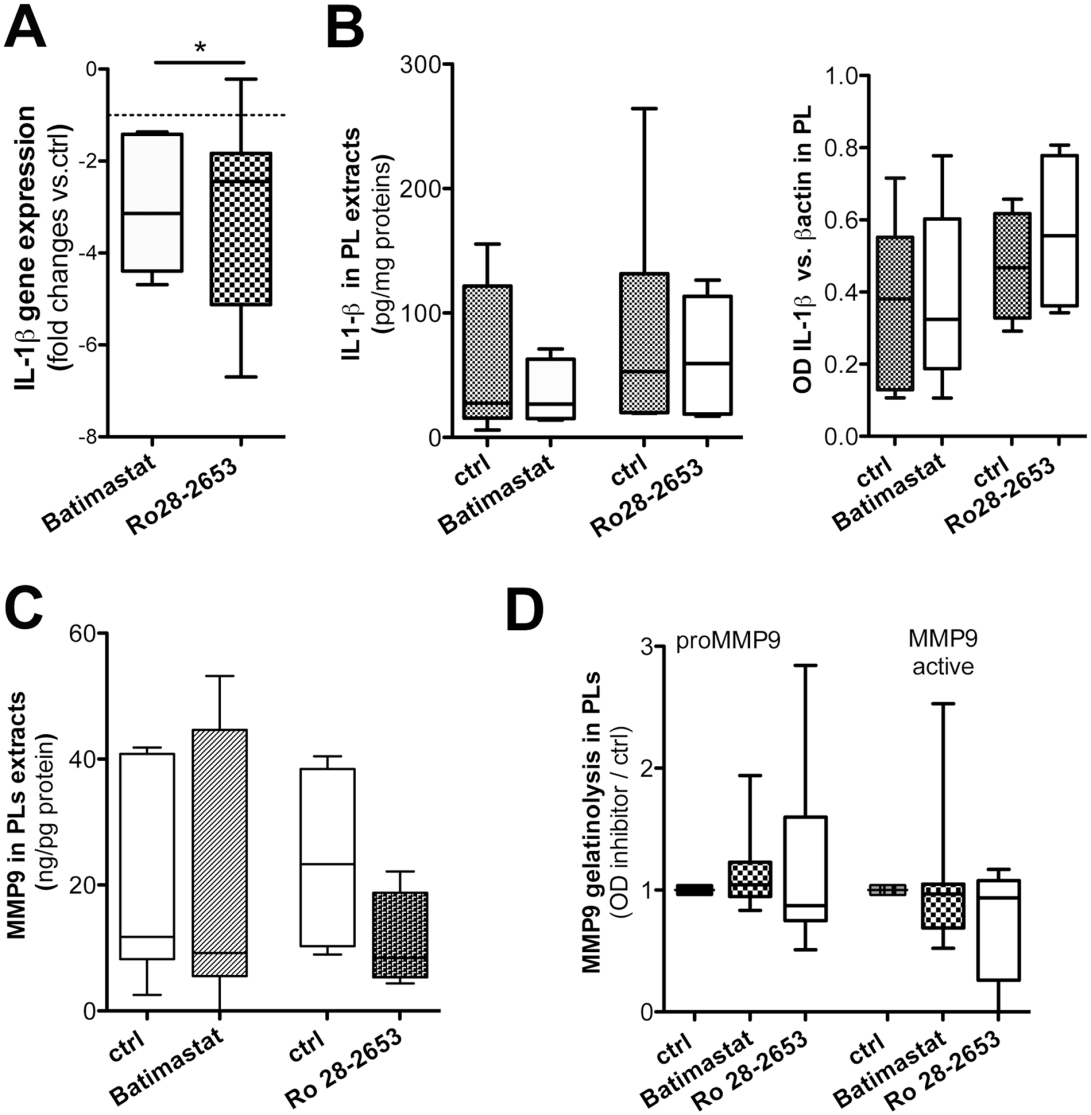
*Ex-vivo* vessel cultures: effect of MMPs antagonists. IL-1β mRNA level changes upon PLs treatment either with Batimastat (n = 12) or Ro28-2653 (n = 13) are shown (**A**). Wilcoxon signed rank test is applied. IL-1β amount in PL extracts (**B**, left) and results from densitometry on proIL-1β expression (**B**, right) are displayed. MMP9 content (**C**) and MMP9–related gelatinolytic activity (**D**) in PL extracts are plotted. Values displayed as boxes are shown with whiskers 5–95 percentile. Significant difference is shown as *p < 0.05.

MMPs pathway blockade was applied to evaluate its power over P2X7 antagonism in affecting IL-1β and the eventual activation of a feedback loop. PLs were treated either with Batimastat, a broad-spectrum MMP inhibitor acting on ERK pathway, or with Ro28-2653, a new inhibitor of unknown mechanism but highly selective for MMP2, MMP9 and MT-1. Batimastat (p = 0.0313) but not Ro28-2653 (p = 0.0625) significantly decreased the IL-1β gene expression (Fig. 6A). MMP inhibitors had no effect on IL-1β content and on proIL-1β protein expression (Fig. 6B), as well as on and P2X7, NLRP3 and caspase-1 (not shown).

Surprisingly Batimastat and Ro28-2653 had no remarkable effects also on MMP9; Ro28-2653 only showed a tendency towards decreasing the MMP9 content (Fig. 6C) and the gelatinolytic activity of MMP9 active (Fig. 6D).

These findings indicated that bona fide MMPs inhibitors when applied to *ex-vivo* PL tissue cultures slightly acted at mRNA level, without relevant post-transcriptional effects.

### Experimental and clinical data comparison

Excluding the relationship between MMP9 and patient’s smoking status mentioned above, no association was found between experimental and clinical data (Supplementary Tables II and III), including demographics.

## Discussion

ATP-triggered P2X7-related secretome^15^ may generate a broad range of effects including unconventional release of the pro-inflammatory leaderless cytokines IL-1β and IL-18^16, 17^, and MMP9 activation and release^7, 18^. In mice P2X7 is involved in endothelial dysfunction and may elicit the endothelial release of IL-1β^19^, which in PLs was enhanced by stimulation of NLRP3 with exogenous ATP^20^. P2X7 has been detected into PLs^11^, as well as on the endothelium of IMA and saphenous vein in the settings of CABG^21^. Therefore a possible role for P2X7 in atherosclerosis in relationship to lesion/activated status and hinting to a connection with MMPs has been proposed^11, 22^.

We found P2X7 at 54 kD as the only isoform detectable in both IMAs and nPLs and the prevalent one in PLs. This functional isoform is the predominant P2X7 transcript in many tissues^23^, is able to reproduce all the full-length –dependent responses excepted apoptosis and large pore formation, and is involved in proliferation through sustaining cells survival or hyperplasia^24^. We surmise that low expression of P2X7 at 54 kD in IMA and nPL is associated to maintained homeostatic proliferation rate, its high expression in PL to the neointima development. In IMAs where native MMP system has been related to absence of occlusive lesions in both native and grafted conditions^25^, the prevention of hyperplasia in long-term culture conditions has also been associated to MMP9 inhibition^15^. Consistently lower MMP9 mRNA level was demonstrated in IMA over occluded popliteal artery^26^, and elevated MMP9 expression likely contributed to structural weakening of advanced carotid lesions^27^. Expression of NLRP3, caspase-1 and IL-1β mRNA was significantly increased in biobank PLs compared to normal arteries^20^. In agreement with these data, our findings delineate an activated molecular pattern for atherosclerotic PLs and a non-activated pattern for morphologically normal nPLs and IMAs. The difference between nPL and PL fragments indicates that activated pattern is restricted to the site of stenosis, a relevant point for locally tailored therapies. The similarity between nPLs and IMAs indicates the involvement of dissimilar susceptibility rather than of different phenotype in the responsivity to atherosclerotic stimuli. Extrapolation of this data to explain IMA’s resistance to atherosclerosis remains speculative.

IL-1β but not MMP9 is associated with PL’s instability/vulnerability and is related to the presence of calcified nodules underneath the fibrous cap, confirming recent data^28^ and supporting the hypothesis of tissue IL-1β as an indicator of high-risk PL and a stimulator for vascular calcification^29^. The absence of relationship between pro-IL-1β and PL stability suggests that active form of IL-1β is the actor in this scenario. High IL-1β could be the product of unbalanced IL1 pathway, exerting both pro/anti- atherogenic effects in atherosclerosis^30^. Targeting IL-1β-related pathway to reduce the cardiovascular risk is a challenging opportunity, but trials with IL-1 blockers are lacking. CANTOS clinical trial, applying Canakinumab monoclonal antibody to block IL-1β interaction with its receptors, is still on-going^31^.

The present work investigated the suitability to act upstream through engagement of P2X7 at the ATP binding site. We demonstrate in *ex-vivo* human vessel tissue cultures the post-transcriptional efficacy of treatment with A740003, a specific P2X7 competitive antagonist, on decreasing IL-1β content, MMP9 content and gelatinolytic activity. IL-1β production and cleavage is a tightly regulated multi-step process and may occur through the activation of P2X7 and NLRP3/caspase-1 by danger signals^32, 33^. P2X7 interaction with NLRP3 scaffold, possibly inducing ion changes and inflammasome activation to generate IL-1β has been described^34^. Post-translational processing of IL-1β requires a secondary stimulus, and ATP levels have been associated with/required for caspase-1 activation and IL-1β release. A740003 was effective on IL-1β, but unable to modulate NLRP3 and caspase-1 in PLs, suggesting a blockage of the pathway upstream the synthesis of pro IL-1β that does not involve NLRP3 transduction apparatus, nor affect the maturation/release of IL-1β. Consistently, decrease of both IL-1β and pro IL-1β after 72 h of treatment with A740003 indicated that IL-1β reservoir was exhausting, and supports the hypothesis that pre-existing IL-1β is released and utilized, but not replaced (see Supplementary material graphical abstract). IL-1β decrease occurred in areas where P2X7 and IL-1β were detected on/nearby cells sharing CD68^33^ and sm22. Intermediate phenotypes between smooth muscle and macrophage-like cells in PLs are known^35^ and our data suggests their importance in the immunopathology of atherosclerosis. Establish whether these cells are smooth muscle cell trans-differentiating in lesional macrophages^36^ requires a dedicated study.

Effectiveness of A740003 on MMP9 supports the implication of IL-1β in MMP9 induction by vascular cells^37, 38^ and the observation that overexpression of MMP9 active by macrophages augmented atherosclerosis progression in mice^2^. The indirect connection between P2X7 and MMP9 through IL-1β indicates that A740003 could play a role in the regulation of MMP9 production aimed to atherosclerosis stabilization. In addition, the relationship between MMP9 modulation and patient’s smoking status suggests the potential benefit of P2X7 antagonist in non and ex- smokers but its unsuitability for smokers, highlighting the role of smoke as risk factor in atherosclerosis^39, 40^.

At difference from A740003, treatment with KN62 variably affected IL-1β and MMP9. A740003 is considered P2X7-selective^41^, KN62 is an ATP non-competitive antagonist active also on calcium/calmodulin–dependent protein kinases, thus it might produce counterbalancing reactions in atherosclerosis settings. No evidence is presently available in support of the other possible explanation to the observed discrepancy, i.e. the interaction of A740003 with a molecule different from P2X7.

In addition, the efficacy of treatment with A740003 open the question whether indirect modulation is more successful than direct specific targeting of MMP9, and if any feedback effect on P2X7-related machinery follows. MMPs inhibitors acted at mRNA level without relevant post-transcriptional effects in *ex-vivo* PLs. Batimastat and Ro28-2653 were chosen based on previous application to vascular disease^42, 43^. Indeed, their capability to inhibit MMP9 was not investigated, nor their effects on human arteries with more than intimal thickening. *Ex-vivo* PLs were end-stage, complex lesions where an on-going plethora of mechanistic processes, including interplay between MMPs and their inhibitors, are possible reasons for the poor efficacy of Batimastat and Ro28-2653. Moreover, we cannot exclude that statins^44^ assumed by patients providing for PLs could hamper the efficacy of MMPs inhibitors, while medications seemed not to interfere with P2X7 antagonism, further supporting the suitability of A740003 as a tool to modulate inflammatory status in atherosclerosis.

### Limitations and future applications

In the absence of animal models fully reflecting human atherosclerosis, the use of *ex-vivo* artery fragments from patients to investigate complex atherosclerotic lesions has 3 main limitations: 1. to be a static model limited in the culture duration, 2. to directly expose more than the luminal surface to mediators, 3. to be unsuitable for silencing experiments, thus hampering extensive mechanistic studies.

To overcome part of these problems in view of clinical applications, we envisage the need of dynamic studies under perfusion and the design of nanoparticles for the *in situ* delivery of P2X7 antagonist to be tested in further studies aimed to design a local targeting therapy.

## Materials and Methods

### Reagents and antibodies

Commercial grade chemicals and reagents for biochemistry were purchased from Sigma-Aldrich, (St. Louis, MO, USA) and Bio-Rad Laboratories (Berkeley, CA, USA), reagents for tissue culture were purchased from Thermo Fisher Life technologies Inc. (Carlsbad, CA USA) and Amersham Hyperfilm ECL were from Amersham Biosciences/GE Healthcare (Little Chalfont, Buckinghamshire, UK).

Primary antibodies: rabbit-anti-P2X7 (AB5581, recognising the extracellular domain of human P2X7, Chemicon, Merck-Millipore group, Temecula, CA, USA), rabbit-anti-NLRP3, rabbit-anti-human caspase-1 (ab1) and polyclonal mouse-anti-human IL-1β (all from Sigma-Aldrich), mouse-anti-human α-Smooth Muscle Actin (α-SMA, 1A4) and goat-anti-human MMP9 (both from R&D systems, Minneapolis, MN, USA). Monoclonal mouse-anti β-Actin-Peroxidase, clone AC-15 (Sigma-Aldrich).

Secondary antibodies: Donkey anti-Rabbit-IgG (H+L) AlexaFluor488, donkey-anti-goat IgG (H+L) AlexaFluor680, donkey anti-Mouse-IgG (H+L) AlexaFluor594 (all from Molecular Probes- Invitrogen), anti-mouse, anti-rabbit and anti-goat IgG peroxidase–conjugated antibodies (all from Sigma-Aldrich).

Drugs: A740003 (Tocris Bioscience, R&D Systems), KN62 and Batimastat (Sigma-Aldrich), Ro28-2653 (kindly provided by Hoffmann-La Roche Inc. after Material Transfer Agreement signature).

Slides and reagents for histology were purchased from BioOptica (Milano, Italy), Fluorsave reagent from Calbiochem (Merck-Millipore group, San Diego, CA).

### Patient’s samples

Patients of both sex (n = 75 screened) submitted to endoarterectomy (TEA) for carotid stenosis >50% by NASCET criteria, within 1 month from a TIA or ischemic stroke homolateral to carotid stenosis (Oxford and TOAST classifications), or asymptomatic will be recruited and carotid artery plaque (PL) specimens collected. Exclusion criteria: TIA or ischemic stroke in patients at risk of cardioembolism, haemorrhagic ictus, posterior circle ictus, stenosis <50%, inflammatory diseases, unrepresentative carotid samples, i.e. missing the atheromasic portions for surgical/mechanical reasons.

Patients of both sex (n = 15) submitted to CABG provided internal mammary artery (IMA) fragments as a surgery waste.

Baseline demographic and clinical data were organized in anonymous database.

The study protocol conformed to the ethical guidelines of the 1975 Declaration of Helsinki and was approved by the ethic committee at San Raffaele Hospital; all patients signed an informed consent.

### *Ex-vivo* tissue culture

*Ex-vivo* cultures of human vascular rings (3–5 mm of thickness) were carried out in Dulbecco’s Modified Eagle Medium with 4.5 g/D-Glucose, L-glutamine, pyruvate, supplemented with 10% low-endotoxin (<0.5 EU/ml) FBS, 1% penicillin/streptomycin in a 5% CO_2_ standard incubator, applying a tissue culture system which preserved vascular features^45^. Vascular rings similar for macroscopic features and wet weight were either untreated or submitted to treatment with drugs applying non-toxic doses previously set up by *in vitro* experiments with PLs–derived primary smooth muscle cells (not shown). A740003 was added to culture medium at 100 μM for 24 h (t1) or 72 h (t3, without medium replacement). KN62 was used at 10 μM for 24 h (t1). Batimastat was used at 10 μM and Ro28-2653 at 20 μM. At the end of treatments, the culture supernatants were harvested and artery rings were rinsed in Dulbecco’s phosphate buffer saline (PBS) and either frozen in isopentane/liquid nitrogen (n = 47, destined to microscopy and protein expression analyses), or immersed in RNA later solution (n = 34, destined to gene expression study). PLs (n = 12) where the atheroma covered a long portion of the carotid length were investigated for both protein and gene expression. PL where the biopsy slightly exceeded the atheroma extension (n = 7) provided also fragments of not atherosclerotic artery (nPL), which allowed performing a partial comparison with IMA tissues.

### Histology and Immunofluorescence

Vascular cryosections (10 μm thick) were stained either with Hematoxylin/Eosin or Movat’s pentachrome to evaluate the vascular morphology following Virmani’s modified AHA classification^46^, or submitted to immunofluorescence. Briefly, acetone- fixed sections were permeabilized with 0.1% triton X-100 in PBS for 10 min, non-specific binding was blocked by 1% BSA in PBS, then slides were labelled with primary antibodies diluted in 1% BSA in PBS. P2X7 (1:200, 2 h, RT), α-SMA (1:500, overnight, 4 °C), CD68 (1:200, 2 h, RT), IL-1β (1:200, overnight, 4 °C), MMP9 (1:200, overnight, 4 °C), caspase-1 (1:100, overnight, 4 °C) were labelled. Upon rinse in PBS, the sections were incubated with appropriate conjugated secondary antibodies (1:500 dilution, 45 min, RT), and then nuclei were stained with 4′,6-diamidino-2-phenylindole (DAPI, 0.2 nmol/L, 10 min, RT). Fluorsave-mounted sections were analysed under Leica TCS SP5 confocal microscope (Leica Microsystems GmbH, Weitzlar, Germany) and 2D free projection max images collected.

### Western blot

Vessel tissues were lysed in RIPA buffer supplemented with complete protease inhibitors cocktail (1:100 dilution) using a TissueRuptor (QIAGEN). Bio-Rad Protein Assay was used for determining the concentration of proteins in tissue extracts. After denaturation in 1% 2-mercaptoethanol-containing sample buffer (5 min, 95 °C) proteins (25 μg) were loaded and resolved on 10% or 12% SDS-PAGE, then transferred onto 0.2 μm nitrocellulose membrane. Non-specific binding was blocked with 5% milk in 0.1% tween20 in PBS, (1 h, RT). The membranes were incubated under gentle shaking overnight, 4 °C either with anti-P2X7 R (1:1500), anti-MMP9 (1:500), anti-IL-1β (1:1000), anti-caspase-1 (1:500), βactin (1:30000, loading control for all samples). After washing, the membranes were submitted to secondary antibodies (1:10000 1 h, RT). Clarity™ western ECL substrate allowed visualization of protein expression on Amersham Hyperfilm ECL by Xograph Imaging System (Compact x4). Optical densitometry was performed with Adobe PhotoshopCS normalizing bands intensity for βactin (http://www.lukemiller.org/journal/2007/08/quantifying-western-blots-without.html).

### ELISA

Human IL-1β and MMP9 were quantified in tissue protein extracts and in culture supernatants by ELISA Quantikine kits (R&D System, Minneapolis, MN, USA) following manufacturers’ instructions. Luminescence measurements were performed on Infinite F200 microplate reader (TECAN Group Ltd, Männedorf, Switzerland). Samples were run at least in duplicate.

### Gel Zymography

Tissue protein extracts and culture supernatants were submitted to gel zymography to evaluate the MMPs gelatinolytic activities. Samples diluted in Laemmli buffer were resolved on 8% SDS-PAGE containing 0.1% gelatine. Gels were washed in 2.5% Triton X-100 at RT on orbital shaker, then they were incubated overnight, 37 °C in 0.05 M TrisHCL pH 8.8, 0.02% NaN_3_, 5 mM CaCl_2_ solution. Staining with Coomassie blue 0.1%, and destaining with 5% methanol and 7% acetic acid solution followed. Areas of digestion were visualized as non-stained regions. Optical densitometry was performed with ImageJ software^11^.

### ATP assay

ATP content was determined in freshly prepared protein extracts and supernatants from PLs (n = 14/treatment) and IMAs (n = 5/treatment) by bioluminescence-based assay (ATP Determination Kit, Molecular Probes, Eugene, OR) according to manufacturers’ instructions. Samples were run in triplicate and luminescence was measured on Mitras LB 940 (Berthold, Bad Wildbad, Germany)^11^.

### RNA isolation and real-time PCR

Tissues in RNA later were homogenized and total mRNA was extracted using RNeasy mini kit (Qiagen, Hilden, Germany). Synthesis of cDNA was carried out by reverse transcription using High Capacity RNA-to-cDNA Kit (Invitrogen, Carlsbad, CA, USA). Quantitative real-time PCR was performed using TaqMan® Universal Master Mix II and Taqman primer/Fam-labelled probe (Hs00175721_m1, Hs00234579_m1, Hs01555410-m1, Hs00918082-m1, Applied Biosystems, Foster City, CA, USA) determining the relative levels of P2X7, MMP9, IL-1β and NLRP3, respectively. All the procedures were carried out according to the manufacturers’ instruction. Samples were run in triplicate, target gene levels were normalized to that of βactin (Hs99999903_m1) mRNA, and relative expression was determined using the ΔCt method. Variation in gene expression in treated tissues vs. untreated was calculated as fold changes^47^.

### Statistical analysis

The D’Agostino and Pearson omnibus test was applied to assess the normality of data distribution. Data from PLs vs. IMA, treated vs. untreated samples were compared by Wilcoxon signed-ranks test, or Mann-Whitney test, or paired t-test with/without Bonferroni’s post-hoc test. Comparison among clinical and experimental data was performed by non-parametric Kruskal-Wallis–test with/without Dunn’s post-hoc test. Correlation between variables was tested by Spearman correlation test. Probability values of <0.05 were considered statistically significant. Prism 5.0 f software was used.

### Data Availability

All data generated or analysed during this study are included in this published article (and its Supplementary Information files).

## Electronic supplementary material

Supplementary data
