# Supplementary material for: P2X7 receptor antagonism modulates IL-1β and MMP9 in human atherosclerotic vessels
Source: Sci Rep. 2017 Jul 7;7:4872. doi: 10.1038/s41598-017-05137-y (PMC5501842; doi:10.1038/s41598-017-05137-y)
Supplement: Supplementary file 1 — Supplementary data [file 41598_2017_5137_MOESM1_ESM.pdf]

Supplementary material

P2X7 receptor antagonism modulates IL-1β and MMP9 in human atherosclerotic vessels

Maria Lombardi † [1], Maria Elena Mantione † [1], Domenico Baccellieri [2], David Ferrara [2],

Renata Castellano [2], Roberto Chiesa [2], Ottavio Alfieri [2], Chiara Foglieni *

Table I. P2X7-related machinery: correlation between vascular gene expression

IMA Number of XY Pairs=11

	Spearman r	95% confidence interval	P value (two-tailed)	correlation significant (alpha=0.05)
**P2X7 vs. MMP9**	0.4312	-0.2470 to 0.8259	0.1826	No
**P2X7 vs. IL-1β**	0.2569	-0.4226 to 0.7515	0.4348	No
**P2X7 vs. NLRP3**	0.7798	0.3198 to 0.9423	0.0064	Yes
**IL-1β vs. MMP9**	0.4818	-0.1861 to 0.8452	0.1375	No
**IL-1β vs. NLRP3**	0.5182	-0.1389 to 0.8585	0.1072	No
**NLRP3 vs. MMP9**	0.2	-0.4706 to 0.7242	0.5574	No

PL (Number of XY Pairs=25)

	Spearman r	95% confidence interval	P value (two-tailed)	correlation significant (alpha=0.05)
**P2X7 vs. MMP9**	0.5308	0.1595 to 0.7705	0.0063	Yes
**P2X7 vs. IL-1 β**	0.4023	-0.003926 to 0.6946	0.0462	Yes
**P2X7 vs. NLRP3**	0.4754	0.08646 to 0.7386	0.0163	Yes
**IL-1β vs. MMP9**	0.6977	0.4074 to 0.8599	0.0001	Yes
**IL-1β vs. NLRP3**	0.6223	0.2899 to 0.8207	0.0009	Yes
**NLRP3 vs. MMP9**	0.7546	0.5030 to 0.8883	< 0.0001	Yes

Table II. Baseline clinical characteristics of patients undergoing carotid endoarterectomy

	(n = 69)
age (years)	70±17 *
sex (M/F)	50/19
% stenosis	74(63-100) **
carotid (dx/sx)	37/32
controlateral operated carotid artery.	18
**Major risk factors (n)**	
pregressed acute myocardial infarction / ICTUS (>6months)	12
hypertension	56
coronary artery diseases / heart failure	12
peripheral artery diseases / cerebral vasculopaties	11
dyslipidemia	32
smocking status (no/yes/ex)	50/10/9
**Medications (n)**	
statins	48
antiaggregants	61
β-blockers	32
ACE inhibitors / sartans	34

* Mean+SD; ** Media (range)

Table III. Baseline clinical characteristics of patients undergoing CABG

	(n = 14)
age (years)	64±19 *
sex (M/F)	12/2
BMI	28.6±3.1
**Major risk factors (n)**	
smocking status (no/yes/ex)	(8/3/3)
positive family history of acute myocardial infarction / ICTUS	2
hypertension	8
dyslipidemia	8
angina	4
pregressed acute myocardial infarction	2
peripheral artery diseases	1
**Medications (n)**	
statins	12
antiaggregants	13
β-blockers	11
ACE inhibitors	6
diuretics	12

*Mean+SD

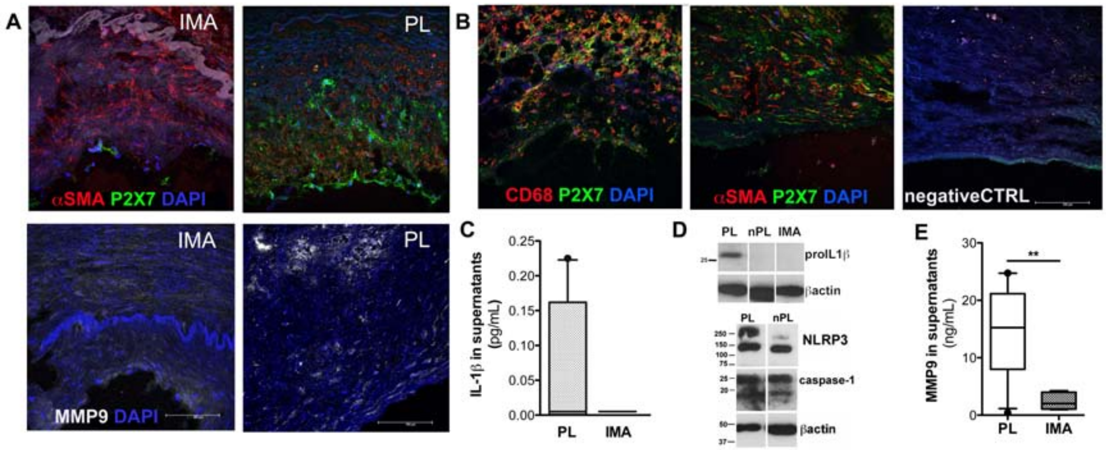

Figure I. ***Characterization of ex-vivo cultures: PL, nPL and IMA***

Confocal microscopy 2D free projection max images from10μm-thick cryosections of PL and IMA display the tissue distribution of P2X7 (green), MMP9 (white) and α-SMA (red) molecules; nuclei were stained with DAPI (blue) (**A**). Confocal images of the same region of intima in three sequential sections from another PL display the localisation of P2X7 (green) in double labelling either with CD68 or α-SMA molecules (red) and the negative control without primary antibodies; nuclei were stained with DAPI (blue) (**B**). Quantification of IL-1β released into culture supernatants of PL and IMA is plotted (**C**). Representative cropped western blots show pro IL-1β in PL, nPL and IMA (**D, top**), NLRP3 and caspase-1 in PL and nPL (**D, bottom**). Quantification of MMP9 content into supernatants of cultured PL and IMA is presented (**E**). Values are shown as boxes with whiskers 5-95 percentile (• indicates outlier); Mann Whitney test is applied. Significant difference is shown as ** p<0.01.

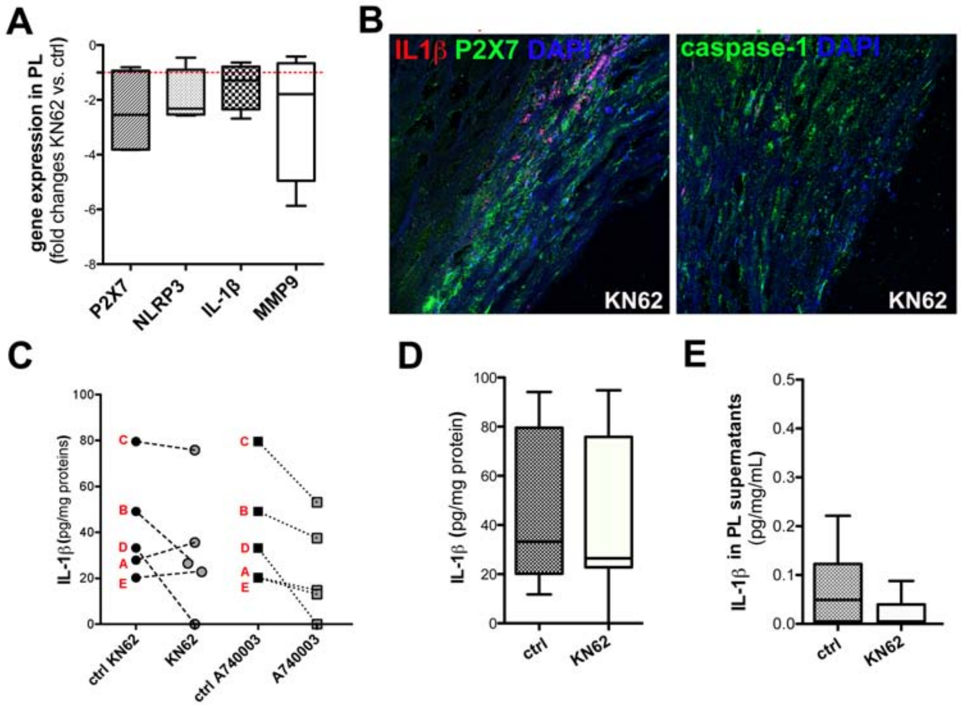

Figure II. ***KN62 effect on ex-vivo cultured PLs***

P2X7, NLRP-3, IL-1β, MMP9 mRNA expression by real-time PCR in PLs after KN62 treatment is shown (**A**). Dotted red line indicates ctrl level. Representative confocal microscopy 2D free projection max images of intima from another fragment of the same PL than in Figure 3C treated with KN62 display the localization of P2X7 (green), IL-1β (red) **(B, left)** and caspase-1 (green) **(B, right)**; nuclei are stained with DAPI (blue). Quantification of IL-1β content in a subset of PLs whose fragments are untreated (ctrl), treated with KN62 or with A740003 is plotted in **C.** Letters indicates fragments from the same vessel. IL-1β content in tissue extracts (**D**) and in culture supernatants (**E**) from PLs (n=8) untreated (ctrl) or treated with KN62 are displayed**.** Values are shown as boxes with whiskers 5-95 percentile.

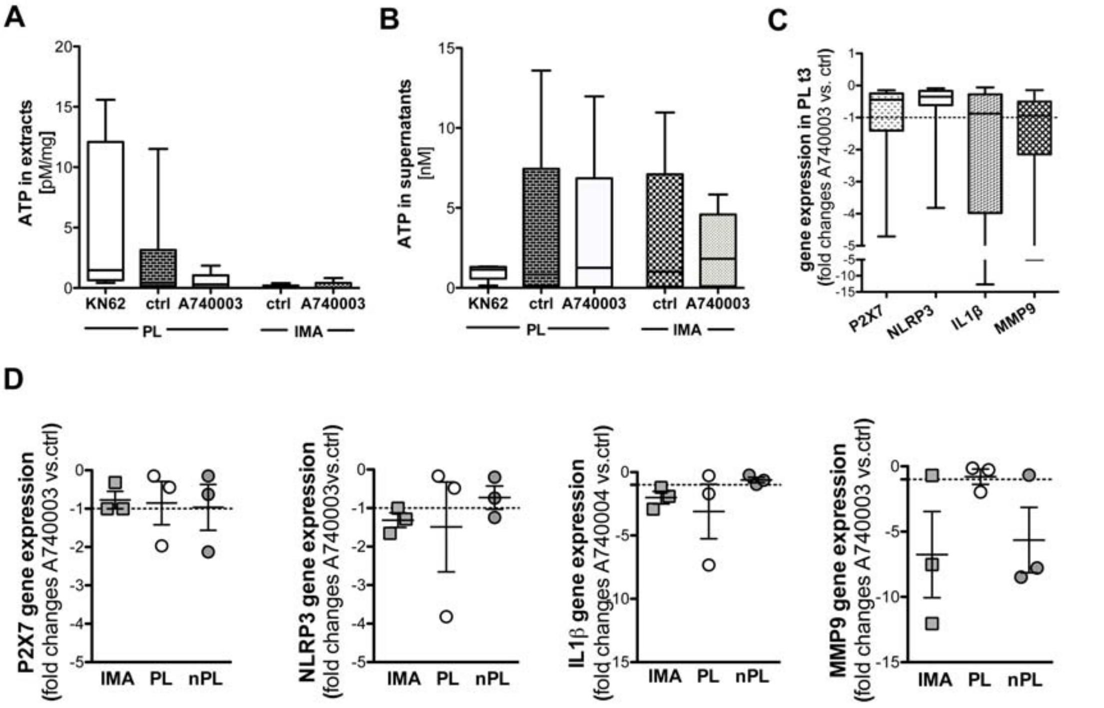

Figure III. ***A740003 effect on ex-vivo cultured arteries***
Quantification of ATP amount into PL and IMA tissue extracts (**A**) and supernatants (**B**) cultured in the absence of treatment (ctrl) or in the presence of treatment with either A740003 or KN62 is presented. The mRNA expression level of P2X7, NLRP-3, IL-1β and MMP9 by real-time PCR in PL after 72h of treatment with A740003 is shown (**C**). Boxes are shown with whiskers 5-95 percentile. The mRNA expression quantified in IMA (n=3) and in a subset of carotid arteries (n=3) with both PL (bearing plaque) and nPL (without plaque) fragments is displayed (**D**). Mean from 3 replicates of the same sample corresponds to each symbol. In scatter dot plots the median with interquartile range is presented. Dotted line indicates ctrl level in **C, D**.

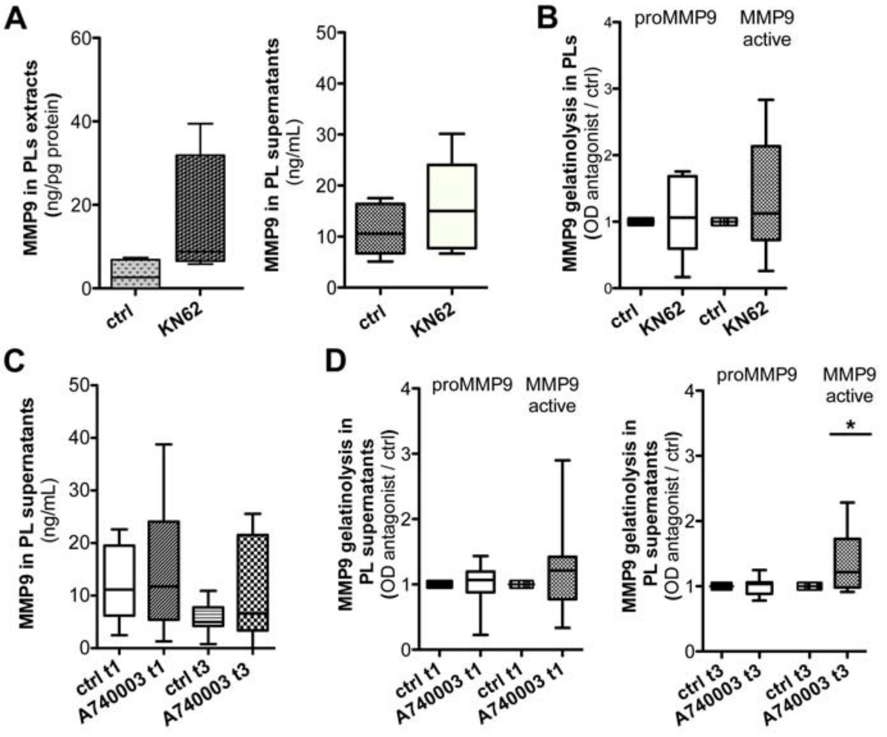

Figure IV. Effects of P2X7 antagonists on MMP9

Effect of treatment with KN62 on MMP9 content (**A**) in PL tissue extracts and supernatants and on MMP9-related gelatinolytic activities in PL tissue extracts **(B)** is shown. MMP9 content (**C)** and gelatinolytic activities **(D)** in supernatant of PL treated with A740003 are presented. Values are shown as boxes with whiskers 5-95 percentile. Significant differences are shown as * $p<0.05$

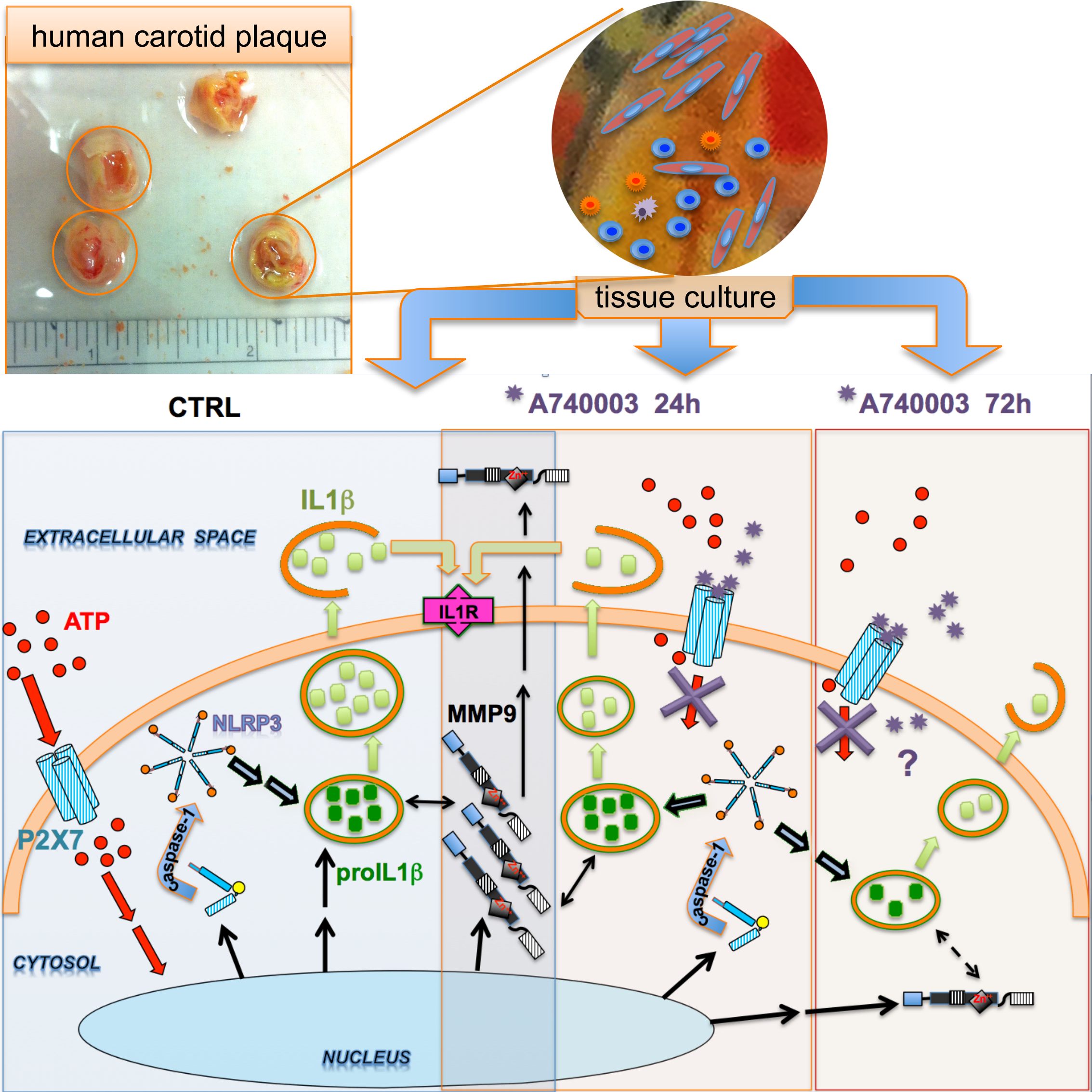

human carotid plaque
tissue culture
CTRL
A740003 24h
A740003 72h
IL1β
EXTRACELLULAR SPACE
IL1R
ATP
NLRP3
MMP9
P2X7
caspase-1
proIL1β
CYTOSOL
NUCLEUS
?
